# Supplementary material for: Dopamine D2 receptor upregulation in dorsal striatum in the LRRK2-R1441C rat model of early Parkinson’s disease revealed by in vivo PET imaging
Source: Sci Rep. 2025 May 7;15:15943. doi: 10.1038/s41598-025-99580-x (PMC12059153; doi:10.1038/s41598-025-99580-x)

Dopamine D2 receptor upregulation in dorsal striatum in the *LRRK2*-R1441C rat model of early Parkinson's disease revealed by *in vivo* PET imaging

Teresa Delgado-Goñi[1,2#], Natalie Connor-Robson[1,3#], Milena Cioroch[1,3], Stephen Paisey[4], Christopher Marshall[4], Emma L. Lane[5], David Hauton[6], James McCullagh[6], Peter J. Magill[1,7], Stephanie J. Cragg[1,3], Clare E. Mackay[1,2], Richard Wade-Martins[1,3], Johannes C. Klein[1,8*].

1. Oxford Parkinson's Disease Centre (OPDC), University of Oxford, Oxford, UK.
2. Department of Psychiatry, University of Oxford, Oxford, UK.
3. Department of Physiology, Anatomy and Genetics, University of Oxford, Oxford, UK.
4. Wales Research & Diagnostic PET Imaging Centre (PETIC), School of Medicine, Heath Park, Cardiff University, Cardiff, Wales, UK.
5. School of Pharmacy and Pharmaceutical Sciences, Cardiff University, Cardiff, Wales, UK.
6. Department of Chemistry, University of Oxford, Oxford, UK.
7. Medical Research Council Brain Network Dynamics Unit, University of Oxford, Oxford, UK.
8. Wellcome Centre for Integrative Neuroimaging, FMRIB, Nuffield Department of Clinical Neurosciences, University of Oxford, Oxford, UK.

# Teresa Delgado-Goñi and Natalie Connor-Robson contributed equally to this work.

SUPPLEMENTARY TABLES

TABLE S1: Average dynamic [^{18}F]FDOPA uptake (net influx rate Ki) in the left and right striatum of animals in the 3 groups investigated. Data represent Mean ± SD. Paired t-Tests used for statistical comparisons.

	[^{18}F]FDOPA Ki (1 x 10^{-2} mL/cm3/min)		
	**Left Striatum**	**Right Striatum**	**p value**
**nTG**	1.3 ± 0.22	1.3 ± 0.24	0.361
**G2019S**	1.3 ± 0.18	1.3 ± 0.21	0.537
**R1441C**	1.3 ± 0.11	1.3 ± 0.13	0.322

TABLE S2: Average cold tracer amount (nmol/kg) injected per animal in each group. Data represent Mean ± SD. Kruskal-Wallis test used for statistical comparisons.

	Average cold tracer injected per animal (nmol/kg)	
	**DOPA**	**Fallypride**
**nTG**	2.91 ± 0.0149	1.15 ± 0.004
**G2019S**	2.34 ± 0.0137	1.07 ± 0.001
**R1441C**	2.79 ± 0.0135	0.95 ± 0.009
**p value**	n.s.	n.s.

TABLE S3: Average dynamic [^{18}F]Fallypride uptake (Distribution volume ratio, DVR) in the left and right striatum of animals in the 3 groups investigated. Data represent Mean ± SD. Paired t-Tests used for statistical comparisons.

	[^{18}F]Fallypride DVR		
	**Left Striatum**	**Right Striatum**	**p value**
**nTG**	15.9 ± 4.02	16.0 ± 4.08	0.853
**G2019S**	16.4 ± 3.24	16.2 ± 3.13	0.189
**R1441C**	20.8 ± 2.51	21.1 ± 1.93	0.263

TABLE S4: Specific activity (GBq/µmol) of tracer injected per animal in each group. Data represent Mean ± SD. Kruskal-Wallis test used for statistical comparisons.

	Specific activity of the tracer calculated at injection (GBq/µmol)	
	**DOPA**	**Fallypride**
**nTG**	426 ± 464.7	623 ± 414.7
**G2019S**	469 ± 622.5	391 ± 171.7
**R1441C**	511 ± 407.1	397 ± 359.6
**p value**	0.615	0.44

SUPPLEMENTARY FIGURES

FIGURE S1: Patlak plot modelling applied to a representative case (1 rat) to assess [18F]FDOPA dynamic uptake in **A)** left and right striatum and **B)** Dorsal and ventral left/right striatum. Region segmentation reduces significantly the CNR affecting the model fitting.

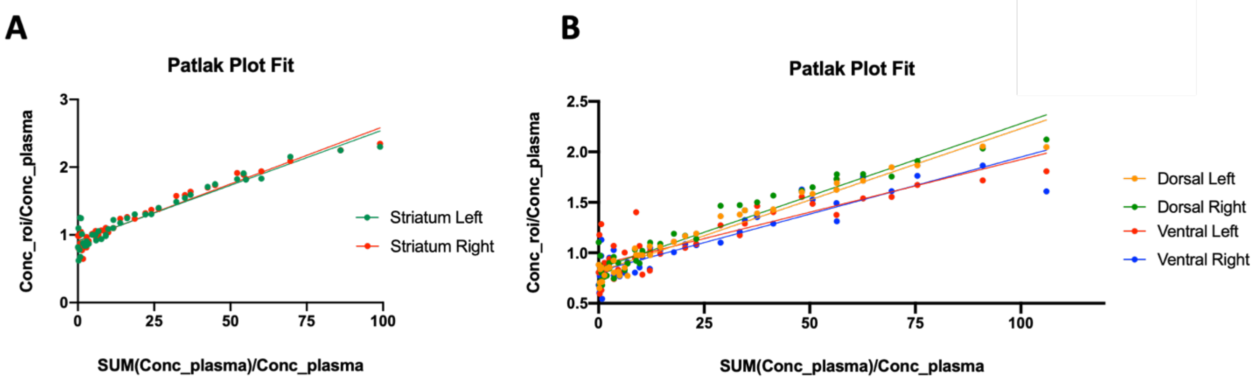

FIGURE S2: Anatomical location of the striatum segmentations used for [^{18}F]Fallypride analysis shown on Paxinos rat brain atlas (coronal and sagittal orientations). Dorsolateral striatum regions are delimited by green lines and ventral striatum regions (nucleus accumbens) are delimited by red lines.

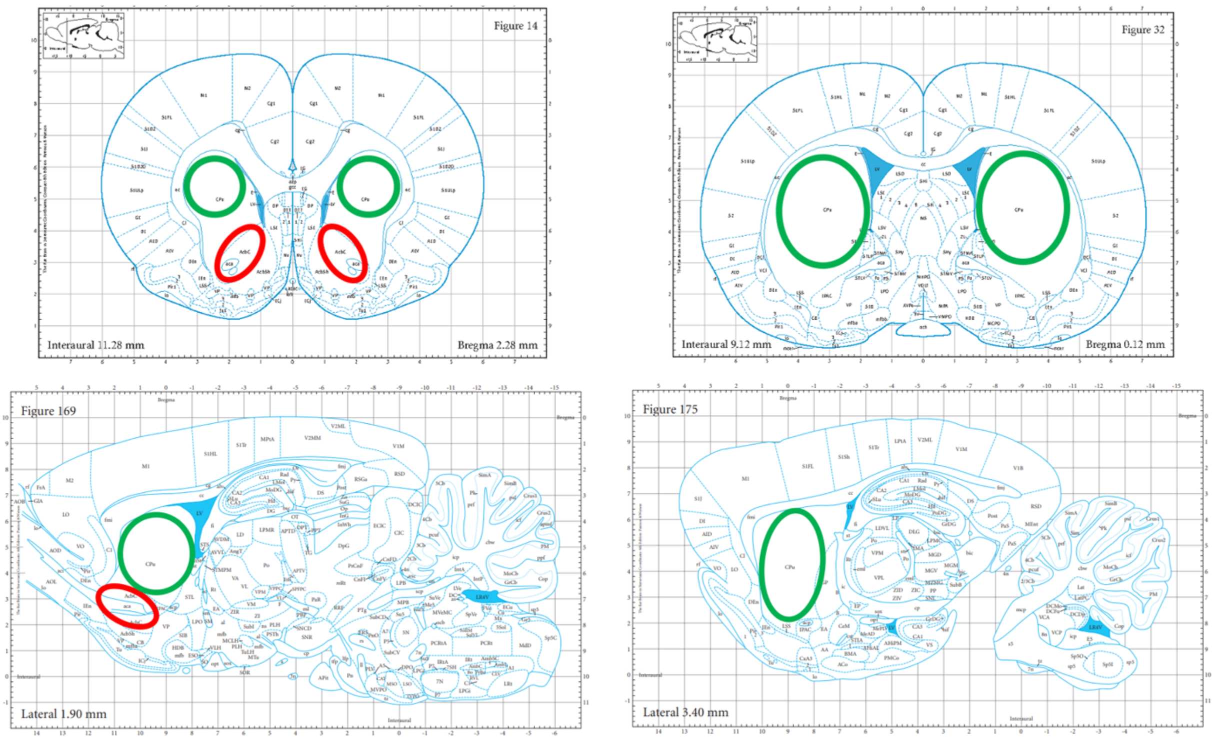

Supplement: Supplementary file 1 — Supplementary Information. [file 41598_2025_99580_MOESM1_ESM.pdf]
